# Isolation and characterization of porcine epidemic diarrhea virus with a novel continuous mutation in the S1^0^ domain

**DOI:** 10.3389/fmicb.2023.1203893

**Published:** 2023-05-18

**Authors:** Xueying Han, Yangkun Liu, Yan Wang, Tiejun Wang, Ning Li, Feng Hao, Lunguang Yao, Kangkang Guo

**Affiliations:** ^1^College of Veterinary Medicine, Northwest A&F University, Shaanxi, China; ^2^Henan Provincal Engineering and Technology Center of Health Products for Livestock and Poultry, School of Life Science and Agricultural Engineering, Nanyang Normal University, Nanyang, China; ^3^Center of Advanced Analysis & Gene Sequencing, Zhengzhou University, Zhengzhou, Henan, China; ^4^Henan Province Engineering Technology Research Center of Animal Disease Control and Prevention, Nanyang Vocational College of Agriculture, Nanyang, Henan, China

**Keywords:** porcine epidemic diarrhea virus, S1^0^ domain, continuous mutation, pathogenicity, isolation

## Abstract

Porcine epidemic diarrhea virus (PEDV), which re-emerged in China in 2010, has caused severe economic losses to the global pig industry. In this study, a PEDV strain, designated PEDV WMB, was isolated from piglets with severe diarrhea on a pig farm in Henan Province of China. Whole-genome sequencing and analysis revealed that the PEDV WMB strain belongs to subtype G2c and has a unique continuous mutation in the S1^0^ antigenic epitope of the S protein. Moreover, the virus-neutralization (VN) test indicated that polyclonal antibodies against the S1^0^ protein of other G1 and G2 strains showed reduced VN reactivity to PEDV WMB. The pathogenicity of PEDV WMB was further investigated in 3 day-old piglets. PEDV infection-related clinical symptoms and morphological lesions were observed and confirmed by histopathological and immunohistochemical examination (IHC). These results illustrated that continuous mutation of the S1^0^ epitope might affect the immunogenicity or pathogenicity of PEDV, providing evidence of the need to monitor the genetic diversity of the virus and develop effective measures to prevent and control PEDV.

## Introduction

Porcine epidemic diarrhea (PED) is a highly contagious and devastating intestinal disease that is characterized by acute diarrhea, vomiting, and dehydration, with high mortality rates of up to 100% in neonatal piglets (Wang et al., 2018). PED was first reported in the United Kingdom in 1978, and it spread to multiple European and Asian countries in the following decades, with sporadic and regional outbreaks (Pensaert and Bouck, 1978; Lin et al., 2016). However, since October 2010, a large-scale outbreak of PED has appeared in many countries in Asia, North America, and Europe (Li et al., 2012). The morbidity and mortality rates in piglets reached 100%, which has brought severe economic losses and hindered the development of the global pig industry (Jung and Saif, 2015; Choudhury et al., 2016).

Porcine epidemic diarrhea virus (PEDV), the causative agent of PED, is an enveloped virus that belongs to the genus *Alphacoronavirus* in the family *Coronaviridae* (Huang et al., 2013). PEDV has a single-stranded positive-sense RNA genome that is approximately 28 kb in length, which consists of seven open reading frames (ORFs): ORF1a, ORF1b, spike (S), ORF3, envelope (E), membrane (M), and nucleoprotein (N) (Song and Park, 2012). As an important surface glycoprotein on PEDV virions, the S protein plays an essential role in the attachment of viral particles to host cell receptors and the induction of neutralizing antibodies (Belouzard et al., 2012; Li et al., 2016; Wang et al., 2019). To date, at least six neutralizing epitope domains have been identified in the S protein: S1^0^ (aa 19–220; Li et al., 2017), S1^A^ (aa 435–485; Chang et al., 2019), the equivalent collagenase domain (COE; aa 499–638; Wang et al., 2017), the SS2 (aa 748–755) and SS6 (aa 764–771) in the S1D region (Sun et al., 2008; Okda et al., 2017), and the C-terminus epitope 2C10 (aa 1,368–1,374) (Cruz et al., 2008). Additionally, the S protein is implicated in virulence, which can be variant readily when receiving immune pressure (Gerber et al., 2014). Therefore, the S gene is divergent and important for developing PEDV vaccines and understanding the genetic variations and relationships between strains.

PEDV strains are divided into the classic G1 group, the variant G2 group, and the recombinant S-INDEL group according to the nucleotide sequences of the S gene (Hanke et al., 2017). The G1 group consists of subgroups G1a and G1b, whereas the G2 group is further classified into subgroups G2a (American and Asian pandemic PEDV strains), and G2b (Chinese non-S-INDEL strains; Wang et al., 2014; Yu et al., 2018). Recently, Guo et al. analyzed the complete genomes of 409 strains from different countries and discovered a new subgroup, G2c (Guo et al., 2019). Recombination analysis showed that the G2c subgroup evolved from a recombination event, with the 5′ part of the S gene acquired from G1a and the remaining genomic regions acquired from G2a. The changes in the S gene, including recombinations, deletions, insertions, and/or mutations, may change the pathogenicity and antigenicity of the variants, leading to the constant emergence of new PEDV mutant strains (Hao et al., 2014; Sun et al., 2018). These findings indicate that the PEDV pandemic strains are undergoing genetic variations, resulting in the genetic diversities of PEDV pandemic strains. With the increased severity and prevalence of PEDV, further investigation of the genetic diversity and molecular characteristics of the S gene of PEDV field strains is urgently needed.

Henan Province is the largest pig-raising region in central China. Since 2010, severe diarrhea outbreaks caused by variant PEDV have been frequently observed in this area, but only partial molecular characteristics of the corresponding PEDV strains were determined (Li et al., 2021). This study successfully isolated a novel PEDV field strain with novel continuous amino acid mutations in the S1^0^ region of the S protein from a pig farm in Henan. Furthermore, the PEDV isolated strains exhibit reduced cross-neutralization activity with the polyclonal antibodies (PAbs) against S1^0^ proteins of other PEDV strains and are pathogenic to 3 day-old piglets. This study provides valuable information to better understand the epidemic characteristics and molecular pathogenesis of PEDV.

## Materials and methods

### Clinical sample collection and treatment

In this study, a total of 32 PEDV-positive intestinal tissues samples were chosen for PEDV isolation. All of these samples were collected from the diseased piglets in commercial pig farms in the Henan Province of China during 2020 to 2022, and examined with a PEDV M gene-based RT-PCR as previously reported (Yu et al., 2018). PEDV-positive intestinal contents were mixed with Dulbecco’s Modified Eagle Medium (DMEM; BI, China) at a ratio of 1:10. The suspension was centrifuged at 5000 × g for 10 min at 4°C, filtered through a 0.22 μm filter, and used as the inoculum for virus isolation.

### Virus isolation and propagation

Vero cells (ATCC CCL-81) were cultured in DMEM supplemented with 10% fetal bovine serum (FBS; Gibco, United States) and 100 U/mL penicillin–streptomycin (Solarbio, China) and seeded in a T25 flask. When they reached about 80% confluence, the cells were washed three times with sterile phosphate-buffered saline (PBS; pH 7.2). An aliquot (1 mL) of a filtered sample, supplemented with 0.3% tryptose phosphate broth (TPB; Sigma-Aldrich, United States) and 10 μg/mL trypsin (Gibco, United States), was inoculated onto Vero cells. After incubation for 1 h at 37°C, the cells were washed three times with PBS. Subsequently, 5 mL maintenance medium (DMEM containing 0.3% TPB, 10 μg/mL trypsin, and 1% penicillin–streptomycin) was added to the cells, and the inoculated cells were cultured at 37°C in 5% CO2. When obvious cytopathic effects (CPEs) were observed in 80–90% of the cells, the flask was subjected to three rounds of freezing and thawing. The cells and culture medium mixture was centrifuged, and the supernatant was stored at −80°C as seed stocks. The isolated PEDV strains, named PEDV WMB, were plaque purified according to methods described in a previous study (Lee et al., 2017). They were passaged serially using the culture supernatant, and the viral titers were determined as the 50% tissue culture infectious dose (TCID50) on Vero cells in a 96-well plate (Sun et al., 2018).

### Indirect immunofluorescence assay

Vero cells in 24-well cell culture plates were mock infected or infected with PEDV WMB at a multiplicity of infection (MOI) of 0.1 for 24 h. The cells were fixed with 4% paraformaldehyde for 30 min at room temperature (RT), washed three times with PBS, and then permeabilized using 0.5% Triton X-100 for 20 min at RT. Subsequently, the cells were blocked with 10% skimmed milk and then successively incubated with mouse anti-PEDV N mAb (Pulike, Luoyang, China) and FITC-conjugated goat anti-mouse IgG (Boster, Wuhan, China). After incubation at RT for 1 h, the cells were washed with PBS, stained with 4′, 6-diamidino-2-phenylindole (DAPI), and examined with a fluorescence microscope (ZEISS, Germany).

### Electron microscopic observation

Vero cells infected with PEDV WMB were collected when obvious CPE was observed. The cells were frozen and thawed three times, and then centrifuged at 6,000 × g at 4°C for 1 h. The supernatant was filtered through a 0.22 μm filter, followed by ultracentrifugation at 30,000 × g for 2 h at 4°C to pellet the PEDV particles. The viral particle pellets were resuspended in PBS, and then negatively stained with 2% phosphotungstic acid and examined with a transmission electron microscope (TEM; Hitachi H-7000FA, Japan). To image virions in the infected Vero cells, the cells were fixed and imaged as described previously (Oka et al., 2014).

### Genome sequencing and phylogenetic analysis

Total RNA from PEDV WMB infected cell cultures was extracted using a Simply P Viral RNA Extraction Kit (BioFlux, China), and cDNAs were then amplified with the RevertAid™ First Strand cDNA Synthesis Kit (Thermo Fisher Scientific, United States). Sixteen pairs of primers were used for PCR to amplify the genomic fragments of the PEDV WMB strain, as described in the previous study. The PCR products were cloned into the pEASY-Blunt Zero vector (Transgene, Beijing, China), and the recombined plasmids were sequenced by Sangon Biological Technology (Shanghai, China). The complete genome of the PEDV strain WMB was assembled according to the sequencing results of 16 overlapping fragments and submitted to the GenBank database. Fifty representative PEDV strains (Supplementary Table S1) were retrieved from the NCBI nucleotide database as reference sequences, including the subgroups G1a, G1b, S-INDEL, G2a, G2b, and G2c. Phylogenetic analysis based on the complete genome and S gene was carried out using the neighbor-joining method in the MEGA 6.06 program. The robustness of the phylogenetic tree was evaluated by bootstrapping using 1,000 replicates. RDP4 was used to evaluate the potential recombination events in PEDV WMB.

### Homology three-dimensional modeling

The 3D structure of the S protein was analyzed using the SWISS-MODEL server based on homology modeling. The S proteins of the PEDV strain WMB and AJ1102 were modeled based on the CV777 S protein. The structural figures were represented by PyMOL software. The server automatically performs a BLASTp search to identify templates for each protein sequence. From the query result, the template protein 6u7k was selected for homology modeling. This is an atomic-resolution structure model of the spike protein from the PEDV with 80.12% sequence identity, which was a reliable score to initiate modeling.

### Virus-neutralization test

The S1^0^ gene of the CV777 vaccine strain (G1 subtype; GenBank accession no. KT323979), the AJ1102 vaccine strain (G2 subtype; GenBank accession no. MK584552), and the isolated WMB strain was codon-optimized and successfully expressed using baculovirus-insect cell expression system. The recombinant proteins were purified with nickel column affinity and then immunized subcutaneously into BALB/c mice to prepare the corresponding PAbs. The PAbs against the S1^0^ protein were inactivated at 56°C for 30 min and then diluted twice starting at 1:25. Then, they were mixed with an equal volume of 200 TCID_50_ of virus, including CV777, AJ1102 or WMB, which were diluted with DMEM supplemented with 10 μg/mL trypsin. After incubating at 37°C for 1 h, 0.1 mL of each mixture was added onto Vero cell monolayers in 96-well plates. After 4 days, CPEs were observed using an inverted microscope (ZEISS, Germany). Each sample was evaluated by three independent experiments.

### Pig challenge experiment

All the pigs used in this study, which were confirmed to be negative for PEDV, TGEV, PoRV, PDCoV, CSFV, and PCV-2 by RT-PCR or PCR detection, were purchased from a commercial pig farm in Henan Province. Ten 3 day-old Landrace piglets were randomly divided into two groups (*n* = 5): PEDV-challenged group and mock control group. Piglets in the mock control group were orally inoculated with 3 mL Vero cell culture supernatant. After the viral challenge, all piglets were evaluated two times daily for mental and physical health status, rectal temperature, and body weight. Anal swabs were collected before inoculation and then every 12 h until death, and fecal consistency was scored as follows: 0 = normal, 1 = pasty, 2 = semiliquid, and 3 = liquid. At 96 h post-infection, all piglets were euthanized and examined for pathology.

During the experiment, the piglets were housed in cages allowing free-walking and were fed commercially purchased cow milk. Animal care and experiments were conducted in accordance with the guidelines of the Ethics Committee of Northwest A&F University (Permit Number: NWSUAF-2021-015). We endeavored to guarantee the best animal welfare.

### Pathological examination

At necropsy, tissue samples including heart, liver, spleen, lung, kidney, intestine, intestinal lymph nodes, and inguinal lymph nodes were collected for viral load measurement. Moreover, portions of the duodenum, jejunum, ileum, cecum, and colon of the piglets from the inoculated and control groups were fixed with 4% paraformaldehyde for histopathological and immunohistochemical (IHC) examinations. Hematoxylin and eosin (HE) staining was performed to visualize pathological changes in intestine tissue samples. An IHC examination was carried out with the mouse anti-PEDV N mAb to visualize the distribution of virus particles in the intestine.

### Quantification of PEDV

Each anal swab was diluted and homogenized in 1 mL of sterile PBS and then centrifuged at 5000 × g at 4°C for 10 min. An aliquot (200 μL) of supernatant was collected for viral RNA extraction. Equal quantities (0.1 g) of tissue samples were homogenized in 1 mL of sterile PBS, and 200 μL of the supernatant was used for RNA extraction. Serum (200 μL) was also used for RNA extraction. The PEDV in different samples was analyzed by absolute real-time quantitative PCR assay with PEDV-specific primers (sense: 5’-TCCGCTAGCTCACGAACAGCCA-3′; antisense: 5’-CAGCATTGCTCTTTGGTGGT-3′). The standard curve was generated using a serially diluted plasmid containing the PEDV N gene. Each sample was tested in triplicate.

### Statistical analysis

All data were expressed as mean ± standard error and analyzed with GraphPad Prism 5 software. Statistical significance between groups was determined using a one-way analysis of variance (ANOVA). Probability (*p*) values of <0.05 and <0.01 were considered statistically significant and highly significant, respectively.

## Results

### Isolation and identification of PEDV WMB

**Figure 1:**
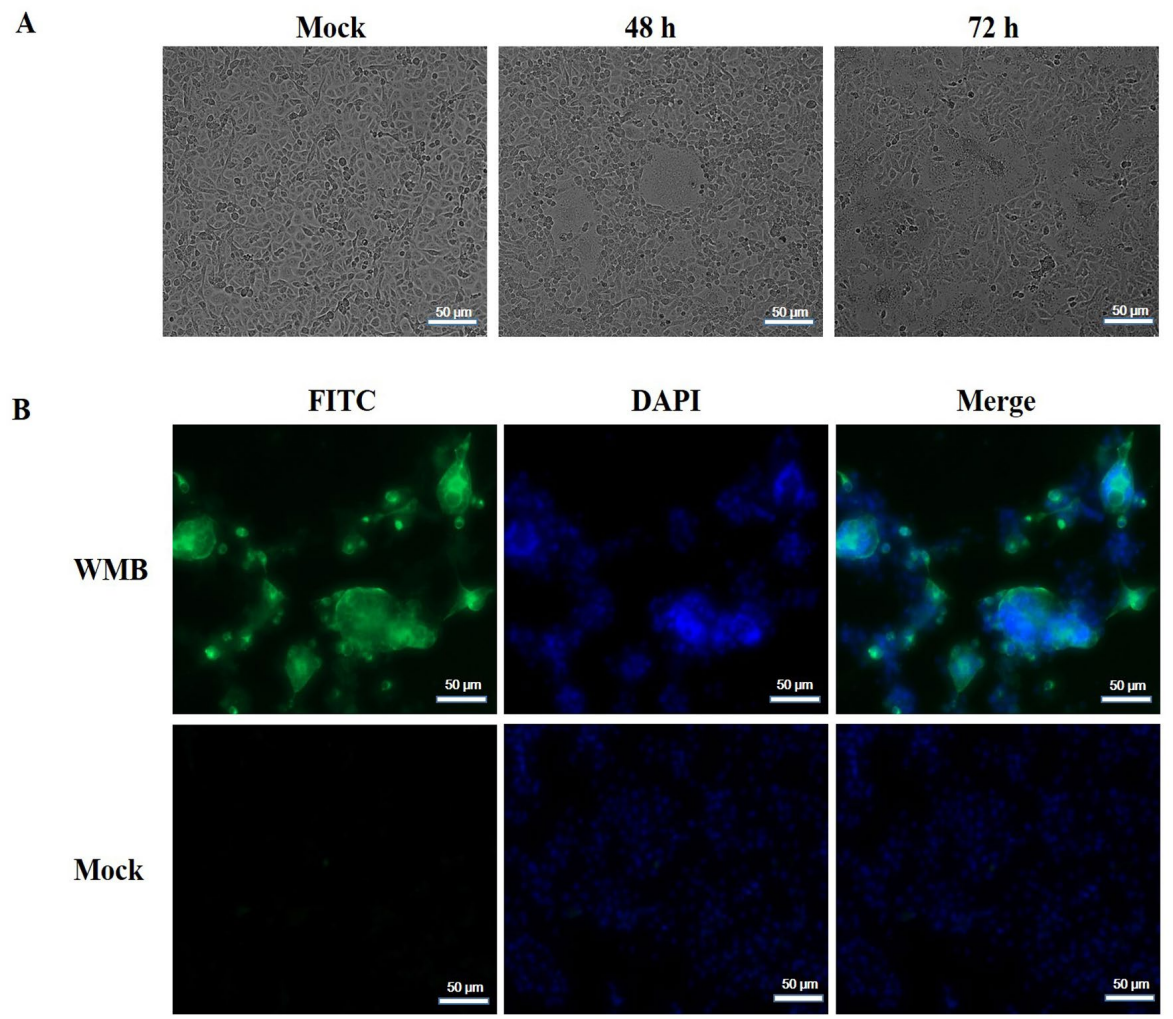
Isolation and characterization of the PEDV WMB strain. **(A)** Vero cells were infected with PEDV WMB, which produced significant CPEs at 48 and 72 h post-infection. **(B)** IFA results of Vero cells infected with PEDV WMB. Vero cells infected with PEDV WMB were stained with the anti-PEDV-N monoclonal antibody as the primary antibody. The FITC-conjugated goat anti-mouse IgG was used as the secondary Ab in IFA. Magnification = 200×.

**Figure 2:**
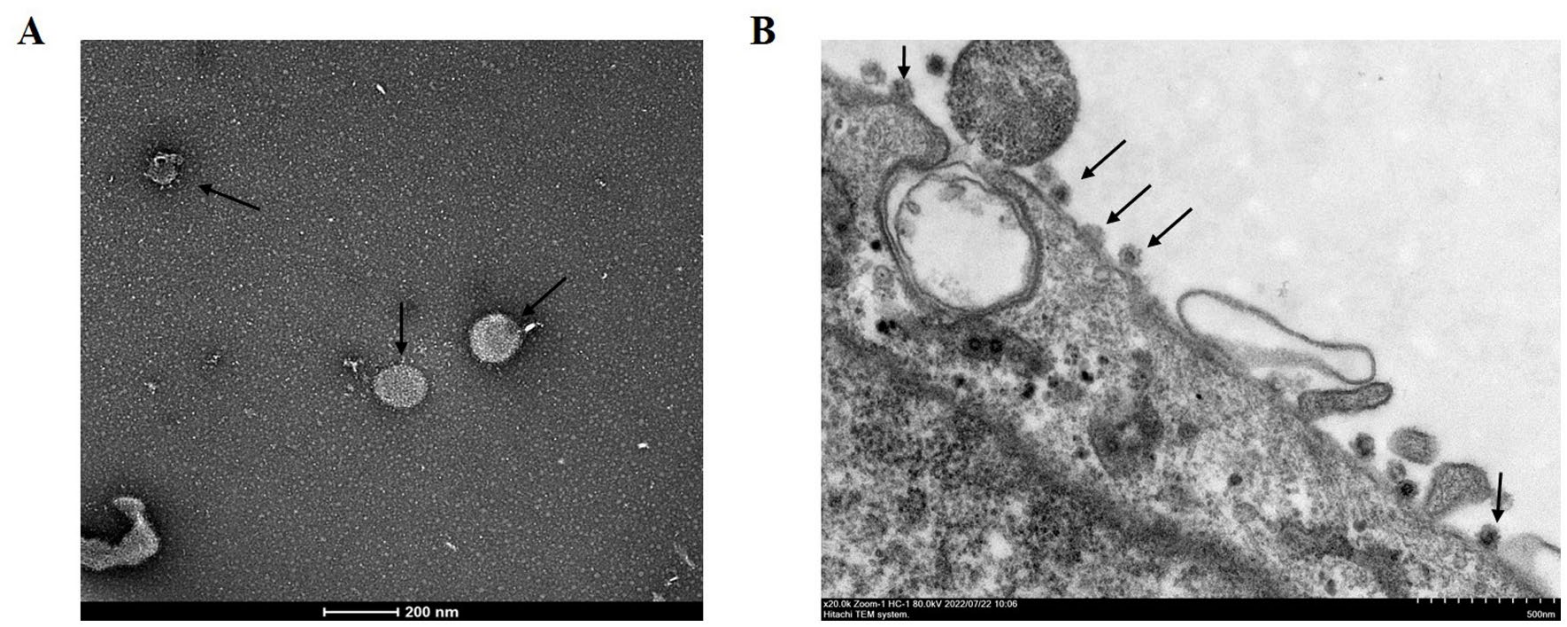
Electron microscopy observation of PEDV virions. **(A)** Arrows show PEDV virions from the cell culture media of Vero cells infected with the PEDV WMB strain. Scale bar = 200 nm. **(B)** Images of a PEDV-infected Vero cell. PEDV particles (arrowheads) on the cell surface of an infected Vero cell, as shown by the arrow. Scale bar = 500 nm.

Vero cells were inoculated with PEDV-positive samples from a 5 day-old piglet and incubated in a maintenance medium at 37°C. A typical CPE, characterized by cell fusion with syncytial and vacuole formation, was observed at 48 h post-inoculation in the second passage. The mock-infected Vero cells showed no signs of CPE (Figure 1A). Furthermore, the viral propagation in Vero cells was confirmed with IFA using anti-PEDV N mAb and DAPI. Specific green fluorescence was observed in the PEDV-infected Vero cells but not in the uninfected Vero cells (Figure 1B). PEDV particles in Vero cell culture media and infected Vero cells were then observed with TEM. The typical coronavirus particles with diameters of about 120 nm were observed in the cell culture media (Figure 2A) and on the surface of PEDV-infected Vero cells (Figure 2B). The isolated PEDV strain was designated as PEDV WMB. After three rounds of plaque purification, the purified viruses were serially passaged for 20 generations in Vero cells. The virus titer of the 5th passage PEDV WMB was 2.14 × 10^6.0^ TCID_50_/mL.

### Genomic and amino acid sequence analyses

**Figure 3:**
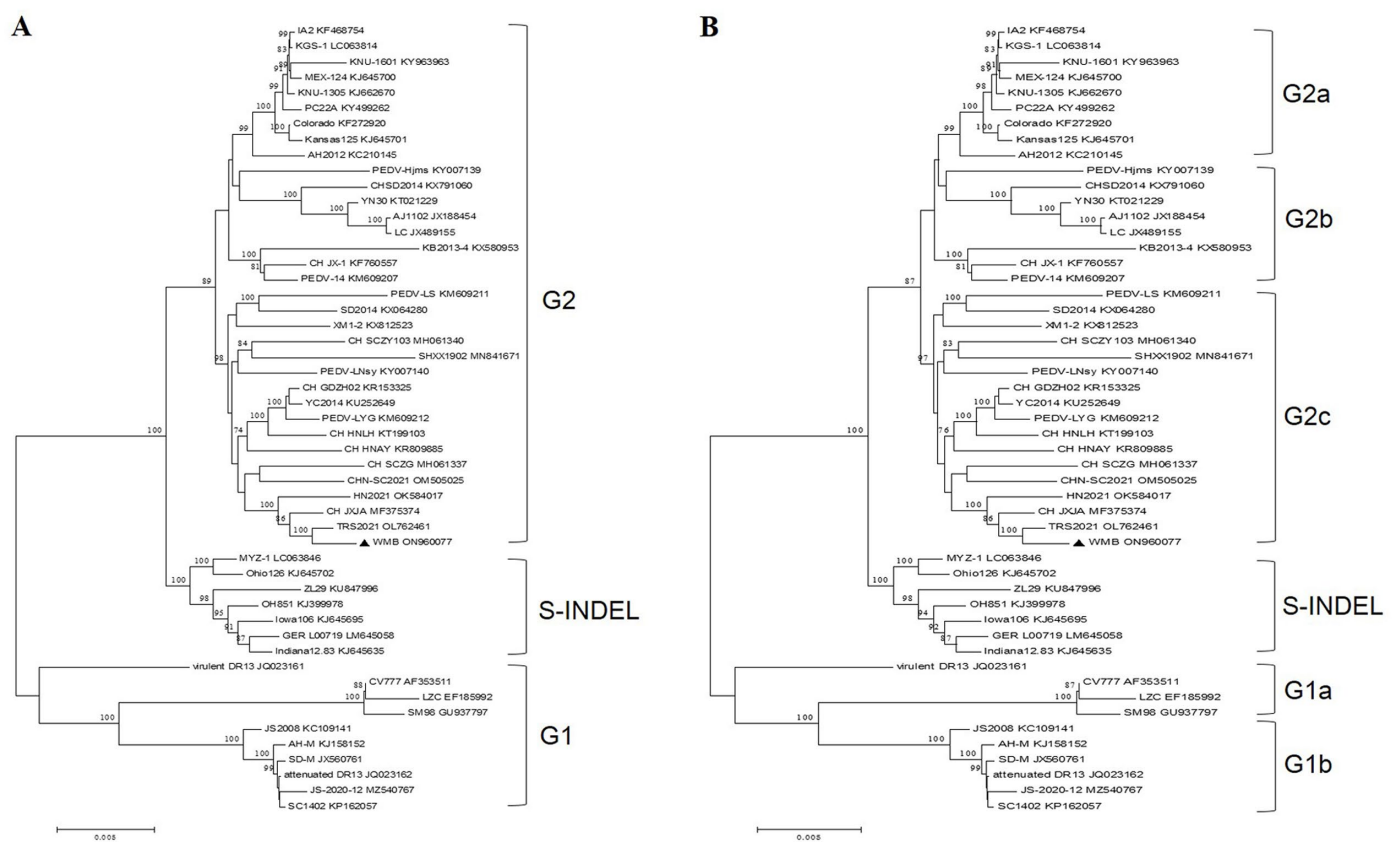
Phylogenetic analysis of PEDV WMB based on nucleotide sequences of the full-length genome **(A)** or the spike gene **(B)**. Phylogenetic trees were constructed with MEGA 6.0 software using the neighbor-joining method. Bootstrap analysis was performed using 1,000 iterations, and the bootstrap value >70% was shown on each branch. The information on reference strains is provided in Supplementary Table S1.

The complete genomic sequence of the PEDV strain WMB was deduced from 16 overlapping cDNA fragments, and submitted to GenBank under the accession number ON960077. The genome of strain WMB is composed of 28,035 nucleotides and includes the following genes: ORF1a (12,354 nt), ORF1b (7,827 nt), S (4,158 nt), ORF3 (675 nt), E (231 nt), M (681 nt), and N (1,326 nt). Based on whole-genome sequence alignments, the PEDV strain WMB shares 96.5–99.7% identity with the 50 reference strains. It has the most similarity with PEDV TRS2021 (99.7% nucleotide identity), while it shares 96.7 and 98.5% nucleotide identity with the vaccine strains CV777 (G1) and AJ1102 (G2), respectively (data not shown). Phylogenetic analysis showed that the PEDV strain WMB was closely related to the G2 subgroup (Figure 3A).

**Figure 4:**
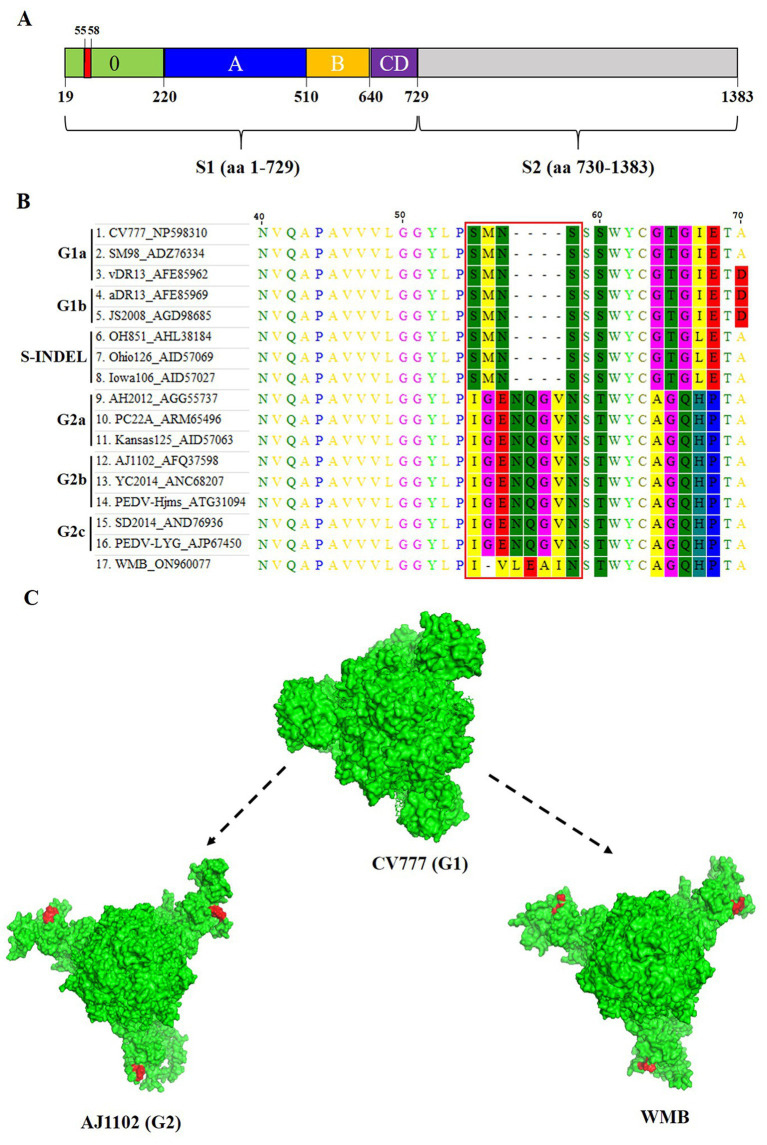
Homology modeling of S protein with the continuous mutation in the S1^0^ region. **(A)** Schematic representation of the PEDV S protein. The different domains in the S1 subunit are colored, with S1^0^ being presented in green, S1^A^ in blue, S1^B^ in orange, and the domains S1^CD^ in purple. The S2 subunit is marked in light gray. **(B)** Sequence alignment of the S protein of different PEDV strains. **(C)** Effects of the continuous mutation in the S1^0^ region of the S protein on the spatial structure predicted by the SWISS-MODEL program. The spatial structures of the S protein were obtained from PEDV WMB, CV777, and AJ1102.

The S gene is an important molecular marker for determining genetic correlations between different PEDV strains and for studying the epidemiological situation. The nucleotide sequence of the S gene of the PEDV WMB is 4,158 nt in length, encoding a predicted S protein containing 1,385 aa. A phylogenetic tree based on the nucleotide sequences of the selected PEDV S genes showed that the PEDV strain WMB fell into the G2c subgroup (Figure 3B). By comparing the aa sequences, a specific continuous mutation in the S1^0^ neutralizing epitope domain (^55^IVLEAI^60^) was found to be unique to the WMB strain (Figures 4A,B). Besides, the WMB S protein structure was predicted using SWISS-MODEL according to the structure of the PEDV CV777 strain in the PDB database (accession code 6u7k). Structure prediction showed that the continuous mutation resulted in the epitope being partially encapsulated (Figure 4C). In addition, recombination detection analysis indicated that no potential recombination events occurred in PEDV WMB (data not shown).

### Cross-virus neutralization test

**Figure 5:**
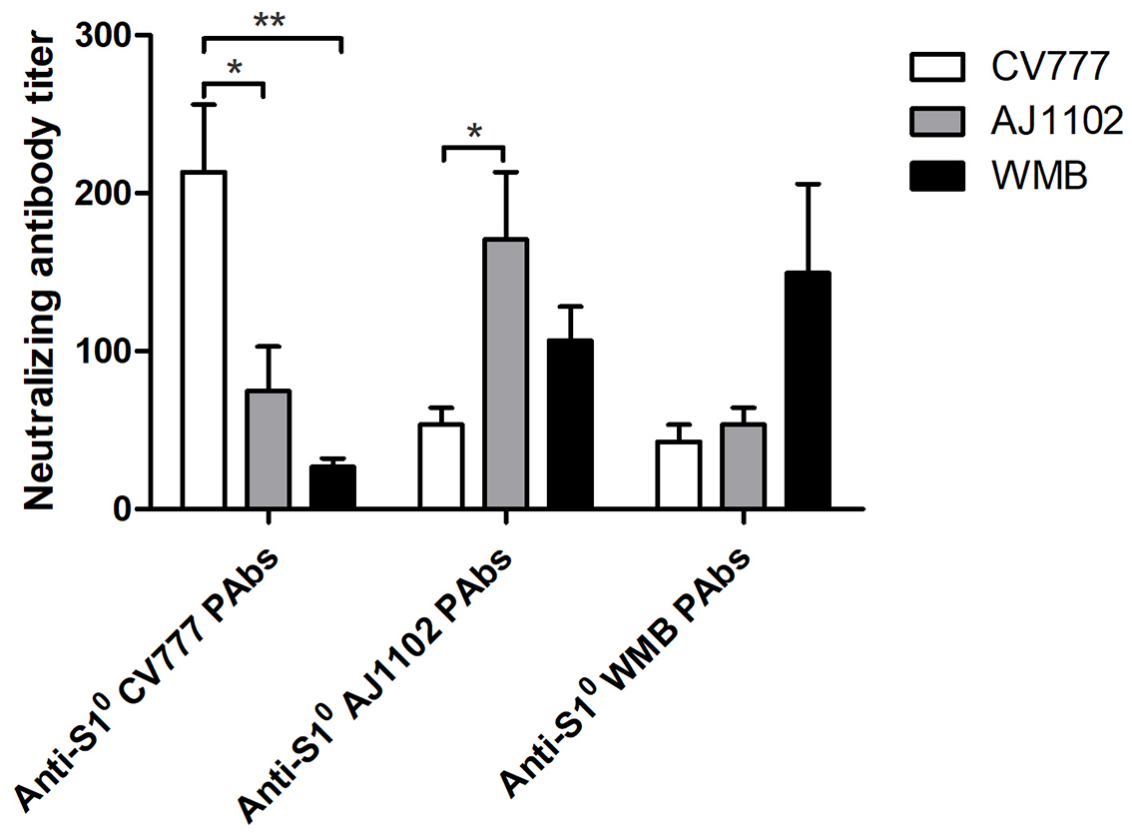
Cross-virus-neutralization between the three PEDV strains and anti-S1^0^ PAbs. Reciprocals of PEDV-neutralizing antibody titers were expressed as the dilution inhibiting PEDV infection by 50%. All data are represented as mean ± SD of three independent experiments. *, *p* < 0.05 were considered as statistically significant and **, *p* < 0.01 were considered as highly significant.

A cross-virus neutralization test was performed to explore whether the continuous mutations in the S1^0^ domain resulted in a change in antigenicity. The results showed that the average titer of CV777 S1^0^ PAb for neutralizing PEDV CV777, PEDV AJ1102, and PEDV WMB was 213, 75, and 27, respectively. The average titer of AJ1102 S1^0^ PAb for neutralizing PEDV CV777, PEDV AJ1102, and PEDV WMB was 53, 171, and 107, respectively. The average titer of WMB S1^0^ PAb for neutralizing PEDV CV777, PEDV AJ1102, and PEDV WMB was 43, 53, and 149, respectively (Figure 5). Therefore, both CV777 PAb and AJ1102 PAb, which belong to the G1 and G2 subtype, respectively, showed reduced VN antibody reactivity to the PEDV strain WMB, indicating that the continuous mutations in the S1^0^ domain may partly alter the profiles of VN antibodies.

### Pathogenicity of PEDV WMB in suckling piglets

**Figure 6:**
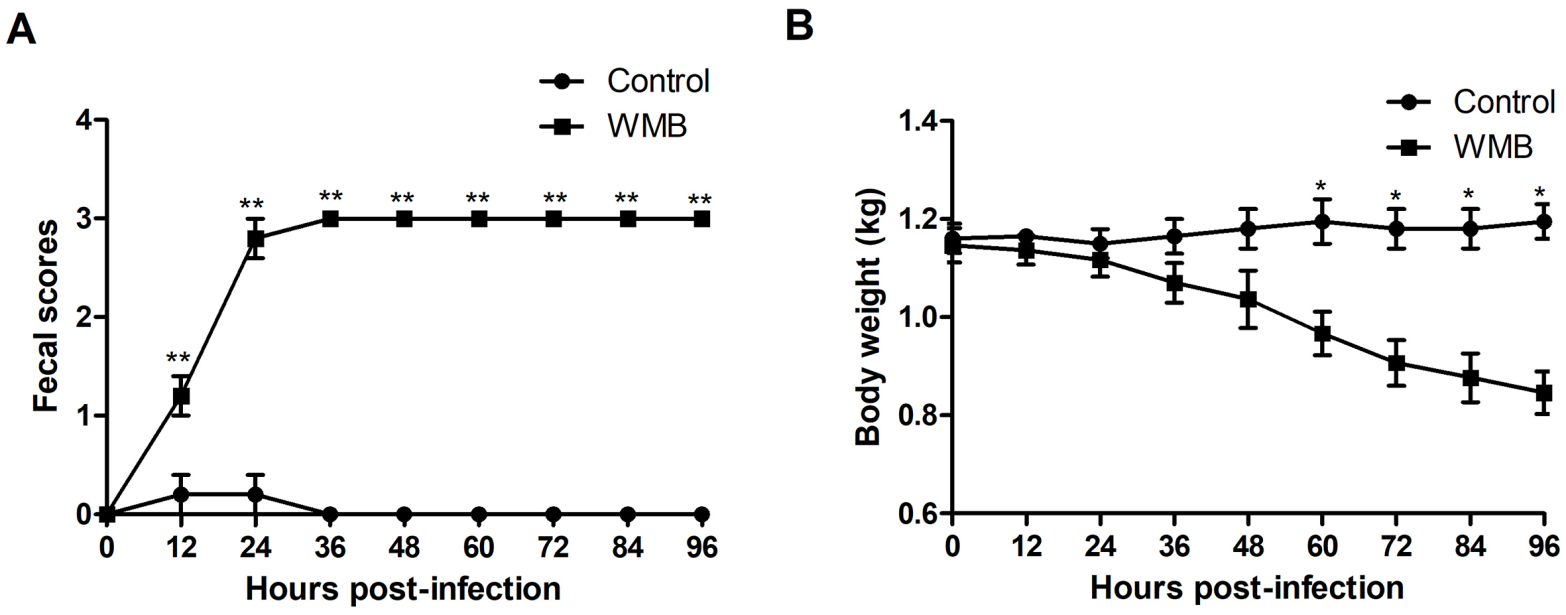
Fecal score and weight of challenged and unchallenged piglets. Data are shown as the mean ± SD. **(A)** Fecal scores of piglets infected with PEDV WMB or DMEM (negative control). **(B)** Weights of piglets infected with PEDV WMB or DMEM (negative control). The asterisk represents the comparison between PEDV WMB group and DMEM group, *p < 0.05 and **p < 0.01.

To evaluate the pathogenicity of the PEDV strain WMB, 3 day-old piglets (*n* = 5) were challenged with the same dose of virus and Vero cell culture supernatant. The piglets inoculated with PEDV WMB exhibited watery diarrhea (fecal score = 3) and vomiting from 24 hpi (Figure 6A); subsequently, they exhibited lethargy, depression, and dehydration, but no challenged piglets died throughout the experiment. Despite the body weights of the challenged piglets gradually decreasing (Figure 6B), the rectal temperatures remained within normal limits. All the piglets from the mock control groups were healthy and active during the experiment.

**Figure 7:**
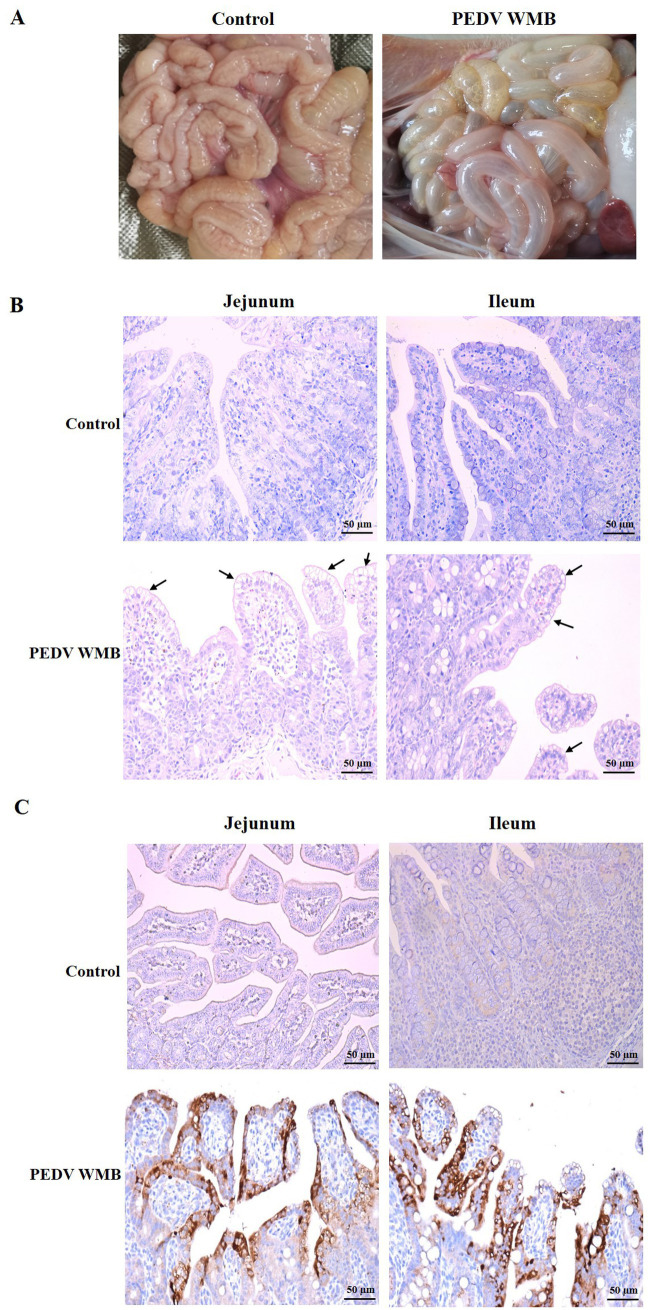
Histopathology and IHC of the small intestines of piglets inoculated with the PEDV WMB strain. **(A)** Necropsy examinations of PEDV WMB infected piglets. **(B)** Histopathological examinations of the intestine of piglets inoculated with PEDV WMB. The arrow indicates that mucosal epithelial cells’ villus atrophy and vacuolar degeneration occurred mainly in the jejunum and ileum. **(C)** Detection of PEDV antigen by IHC analysis. PEDV-specific antigens (brown stain) were mainly distributed in challenged pigs’ jejunum and ileum villus epithelial cells.

All piglets were euthanized at 96 hpi, and the necropsy examinations were performed immediately after the death of the piglets. As shown in Figure 7A, the small intestines of the challenged piglets were thin-walled, clearly transparent, gas-distended, and filled with yellow watery contents, while no intestinal lesions were observed in the uninfected piglets. HE staining showed lesions characterized by apparent atrophy of intestinal villi and vacuolar degeneration of mucosal epithelial cells in the jejunum and ileum of challenged pigs (Figure 7B). Consistent with the histopathological results, the IHC analysis revealed that PEDV-specific antigens (brown stain) were mainly distributed in the jejunum and ileum villus epithelial cells of challenged piglets (Figure 7C).

### Virus distribution and fecal shedding

**Figure 8:**
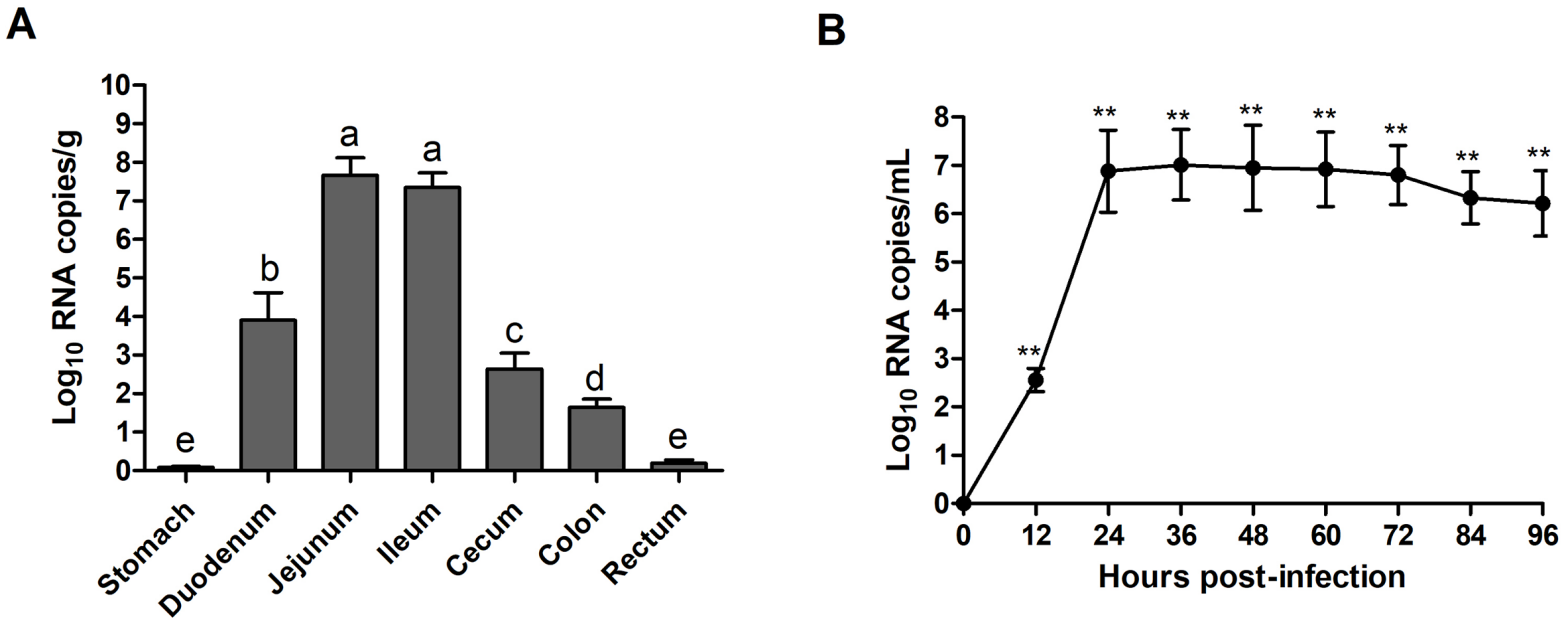
Fecal viral shedding and virus distribution in PEDV WMB-inoculated piglets. **(A)** Virus shedding in rectal swabs of PEDV WMB-inoculated piglets. Different letters indicate significant difference (*p* < 0.05) between groups. **(B)** Virus distribution at 96 hpi in PEDV WMB-inoculated piglets. The asterisk represents the comparison between PEDV WMB group and DMEM group, **p < 0.01.

RT-qPCR was used to evaluate the viral distribution and shedding in challenged piglets’ intestinal tissues and feces. The results showed that all piglets in the control group were negative for PEDV, while high levels of viral RNA were identified in the duodenum (7.94 × 10^3^ RNA copies/g), jejunum (4.57 × 10^7^ RNA copies/g), and ileum (2.26 × 10^7^ RNA copies/g) at 96 hpi (Figure 8A). In addition, all piglets in the experimental group exhibited high levels (3.63 × 10^2^–1.02 × 10^7^ RNA copies/mL) of virus shed in feces from 12 to 96 hpi (Figure 8B). In feces, the viral load reached the highest level at 36 hpi, and then declined slightly at 72 hpi.

## Discussion

Since the emergence of highly pathogenic PEDV variants in October 2010, the PEDV variant G2 has become the major epidemic strain in China, which has seriously affected the development of the swine industry (Wen et al., 2018). Although inactivated and attenuated PEDV vaccines based on the classical CV777 strain have been widely used in the Chinese pig population, PED outbreaks with an 80–100% piglet mortality rate still occurred in the vaccinated herds, indicating that vaccines derived from classical strains cannot provide effective immune protective responses against the currently epidemic strains (Ayudhya et al., 2012; Sung et al., 2019). Furthermore, genome analysis indicates that compared with classic G1 strains, G2 epidemic strains have various amino acid mutations mainly located in the S1 protein (Su et al., 2020). These mutations may alter the viral pathogenicity and antigenicity, resulting in a poor immune response to vaccination and posing a huge challenge to the prevention and control of PED in China (Wang et al., 2016). To deal with the complex genetic and antigenic diversity of PEDV isolates, it is necessary to monitor and analyze emerging PEDV epidemic strains. For this purpose, a PEDV strain was successfully isolated from the clinical sample of the small intestine and serially propagated in Vero cells for up to 20 passages. This PEDV strain, named PEDV WMB, was confirmed with typical CPE, IFA, and TEM. Furthermore, after plaque purification, the viral titer of passage 20 was 1.50 × 10^7^ TCID_50_/mL, indicating that this PEDV isolate was highly adapted to Vero cells. To better understand the biological characteristics of PEDV WMB, we then characterized the full genome and pathogenicity of this new strain in this study.

According to the sequence comparison and phylogenetic analysis based on the full genome, all the referenced PEDV strains in this study share high nucleotide identities. However, the new PEDV strain WMB, belonging to subgroup G2c, is more similar to the strains circulating in the United States than to the Chinese strains. The S gene is an important molecular marker for studying the genetic relationships among different PEDV strains (Lin et al., 2017), and the S protein of PEDV might be highly associated with the pathogenicity and virulence of the virus. In this study, a novel continuous mutation in the S1^0^ region of the S protein in PEDV was identified, resulting in a change in the spatial structure of the S1^0^ region. Although the S1^0^ region is considered the most diverse region between the pandemic and S-INDEL strains (Lin et al., 2016; Chen et al., 2019), none of the currently isolated PEDV strains has been reported to possess multiple amino acid continuous mutations in the S1^0^ region. As the S1^0^ region is a potent neutralizing epitope, mutations in this region may have arisen in some variants that escape immune surveillance. The VN test performed in this study suggested that both CV777 PAbs and AJ1102 PAbs exhibited reduced VN antibody reactivity to the PEDV strain WMB. Consistent with this result, a previous study found that S1^0^ antibodies effectively neutralized only closely homologous non-S-Indel strains (Li et al., 2017). This strategy of viruses to escape immune surveillance imposes a hurdle to vaccine development and needs to be further investigated in future research.

The pathogenicity of PEDV WMB was then confirmed in 3 day-old neonatal piglets. Although clinical observations showed that all piglets inoculated with PEDV WMB exhibited typical clinical signs of PEDV infection, including watery diarrhea and vomiting, their appetite was still maintained, and no deaths occurred over the course of the experiment. Severe intestinal lesions, mainly in the distal jejunum and ileum, were observed in the PEDV-challenged pigs at necropsy, consistent with the histopathological results. These findings indicated that the PEDV strain WMB is pathogenic to piglets, but the disease severity was less than those induced by the highly virulent G2 group strain (Thomas et al., 2015; Madson et al., 2016; Langel et al., 2019). A previous study showed that S INDELs and the associated single-nucleotide polymorphisms (SNPs) in the hypervariable S1 region of the S gene might associate with a less virulent phenotype (Vlasova et al., 2014), indicating that the PEDV strain WMB might be attenuated. Whether these mutations affect viral virulence and immunity needs further study using infectious cDNA techniques.

In summary, a novel Chinese genotype G2c PEDV strain WMB, with unique continuous mutations in the S1^0^ region of the S gene, was successfully isolated from clinical samples. Furthermore, the PEDV strain WMB appeared to be enteropathogenic and caused severe intestinal disease in 3 day-old piglets. Thus, this study provides a candidate PEDV strain for vaccine development. Together, these findings suggest that comprehensive epidemiological investigation is necessary to determine the prevalence of this type of variant in China, and efficient vaccines against these strains should be developed.

## Data availability statement

The datasets presented in this study can be found in online repositories. The names of the repository/repositories and accession number(s) can be found in the article/Supplementary material.

## Ethics statement

The animal study was reviewed and approved by Animal Welfare and Ethics Committee of Northwest A&F University Northwest A&F University.

## Author contributions

XH and YL designed the study. XH, YL, and YW performed the experiments. TW, NL, and FH collected samples and analyzed the data. YL, XH, LY, and KG wrote and revised the manuscript. All authors contributed to the article and approved the submitted version.

## Funding

This work was financially supported by the National Natural Science Foundation of China (32002302, 31870917, and 32272997), Scientific and Technological research project of Henan province (202102110097 and 222102110260) and Shaanxi Special Plan for the Animal Epidemic Prevention Fund Project (20220600180407432).

## Conflict of interest

The authors declare that the research was conducted in the absence of any commercial or financial relationships that could be construed as a potential conflict of interest.

## Publisher’s note

All claims expressed in this article are solely those of the authors and do not necessarily represent those of their affiliated organizations, or those of the publisher, the editors and the reviewers. Any product that may be evaluated in this article, or claim that may be made by its manufacturer, is not guaranteed or endorsed by the publisher.
