# Supplementary material for: Isolation and characterization of porcine epidemic diarrhea virus with a novel continuous mutation in the S10 domain
Source: Front Microbiol. 2023 May 18;14:1203893. doi: 10.3389/fmicb.2023.1203893 (PMC10232790; doi:10.3389/fmicb.2023.1203893)
Supplement: Supplementary file 1 [file Table_1.DOCX]

Supplementary Material

## Supplementary Table S1. PEDV strains used in this study.

| **Strains** | **Accession No.** | **Countries** | **Collection date** | **Genotype** |
| --- | --- | --- | --- | --- |
| CV777 | AF353511.1 | Belgium | 1978 | G1a |
| SM98 | GU937797.1 | South Korea | 1998 | G1a |
| vDR13 | JQ023161.1 | South Korea | 1999 | G1a |
| LZC | EF185992.1 | China | 2006 | G1a |
| aDR13 | JQ023162.1 | South Korea | 2003 | G1b |
| JS2008 | KC109141.1 | China | 2008 | G1b |
| AH-M | KJ158152.1 | China | 2011 | G1b |
| SD-M | JX560761.1 | China | 2012 | G1b |
| SC1402 | KP162057.1 | China | 2014 | G1b |
| JS-2020-12 | MZ540767.1 | China | 2020 | G1b |
| Indiana12.83 | KJ645635.1 | USA | 2013 | S-INDEL |
| Iowa106 | KJ645695.1 | USA | 2013 | S-INDEL |
| MYZ-1 | LC063846.1 | Japan | 2013 | S-INDEL |
| OH851 | KJ399978.1 | USA | 2014 | S-INDEL |
| GER L00719 | LM645058.1 | Germany | 2014 | S-INDEL |
| Ohio126 | KJ645702.1 | USA | 2014 | S-INDEL |
| ZL29 | KU847996.1 | China | 2015 | S-INDEL |
| AH2012 | KC210145.1 | China | 2012 | G2a |
| KGS-1 | LC063814.1 | Japan | 2013 | G2a |
| KNU-1305 | KJ662670.1 | South Korea | 2013 | G2a |
| Colorado | KF272920.1 | USA | 2013 | G2a |
| IA2 | KF468754.1 | USA | 2013 | G2a |
| MEX-124 | KJ645700.1 | Mexico | 2014 | G2a |
| Kansas125 | KJ645701.1 | USA | 2014 | G2a |
| PC22A | KY499262.1 | USA | 2015 | G2a |
| KNU-1601 | KY963963.1 | South Korea | 2016 | G2a |
| LC | JX489155.1 | China | 2011 | G2b |
| PEDV-14 | KM609207.1 | China | 2011 | G2b |
| AJ1102 | JX188454.1 | China | 2012 | G2b |
| KB2013-4 | KX580953.1 | China | 2013 | G2b |
| YN30 | KT021229.1 | China | 2013 | G2b |
| CH JX-1 | KF760557.1 | China | 2013 | G2b |
| CHSD2014 | KX791060.1 | China | 2014 | G2b |
| PEDV-Hjms | KY007139.1 | China | 2015 | G2b |
| YC2014 | KU252649.1 | China | 2014 | G2c |
| PEDV-LS | KM609211.1 | China | 2014 | G2c |
| SD2014 | KX064280.1 | China | 2014 | G2c |
| PEDV-LYG | KM609212.1 | China | 2014 | G2c |
| CH GDZH02 | KR153325.1 | China | 2014 | G2c |
| HNAY | KR809885.1 | China | 2015 | G2c |
| PEDV-LNsy | KY007140.1 | China | 2015 | G2c |
| CH HNLH | KT199103.1 | China | 2015 | G2c |
| XM1-2 | KX812523.1 | China | 2016 | G2c |
| CH SCZG | MH061337.1 | China | 2017 | G2c |
| SCZY103 | MH061340.1 | China | 2017 | G2c |
| CH JXJA | MF375374.1 | China | 2017 | G2c |
| SHXX1902 | MN841671.1 | China | 2019 | G2c |
| TRS2021 | OL762461.1 | China | 2021 | G2c |
| HN2021 | OK584017.1 | China | 2021 | G2c |
| CHN-SC2021 | OM505025.1 | China | 2021 | G2c |
